# Transcripts of Anthocyanidin Reductase and Leucoanthocyanidin Reductase and Measurement of Catechin and Epicatechin in Tartary Buckwheat

**DOI:** 10.1155/2014/726567

**Published:** 2014-01-27

**Authors:** Yeon Bok Kim, Aye Aye Thwe, YeJi Kim, Xiaohua Li, Jin Woong Cho, Phun Bum Park, Mariadhas Valan Arasu, Naif Abdullah Al-Dhabi, Sun-Ju Kim, Tastsuro Suzuki, Kwang Hyun Jho, Sang Un Park

**Affiliations:** ^1^Department of Crop Science, Chungnam National University, 99 Daehak-ro, Yuseong-gu, Daejeon 305-764, Republic of Korea; ^2^Department of Bioscience and Biotechnology, University of Suwon, San 2-2 Wauri Bongdameup, Hwasung 445-743, Republic of Korea; ^3^Department of Botany and Microbiology, Addiriyah Chair for Environmental Studies, College of Science, King Saud University, P.O. Box 2455, Riyadh 11451, Saudi Arabia; ^4^Department of Bio-Environmental Chemistry, Chungnam National University, 99 Daehak-ro, Yuseong-gu, Daejeon 305-764, Republic of Korea; ^5^Hokkaido Agricultural Research Center, National Agriculture and Food Research Organization, Sapporo 062-8555, Japan; ^6^Department of Business Administration, Sahmyook University, Hwarangro 815, Nowon-gu, Seoul 139-742, Republic of Korea

## Abstract

Anthocyanidin reductase (ANR) and leucoanthocyanidin reductase (LAR) play an important role in the monomeric units biosynthesis of proanthocyanidins (PAs) such as catechin and epicatechin in several plants. The aim of this study was to clone ANR and LAR genes involved in PAs biosynthesis and examine the expression of these two genes in different organs under different growth conditions in two tartary buckwheat cultivars, Hokkai T8 and T10. Gene expression was carried out by quantitative real-time RT-PCR, and catechin and epicatechin content was analyzed by high performance liquid chromatography. The expression pattern of ANR and LAR did not match the accumulation pattern of PAs in different organs of two cultivars. Epicatechin content was the highest in the flowers of both cultivars and it was affected by light in only Hokkai T8 sprouts. ANR and LAR levels in tartary buckwheat might be regulated by different mechanisms for catechin and epicatechin biosynthesis under light and dark conditions.

## 1. Introduction

**Figure 1:**
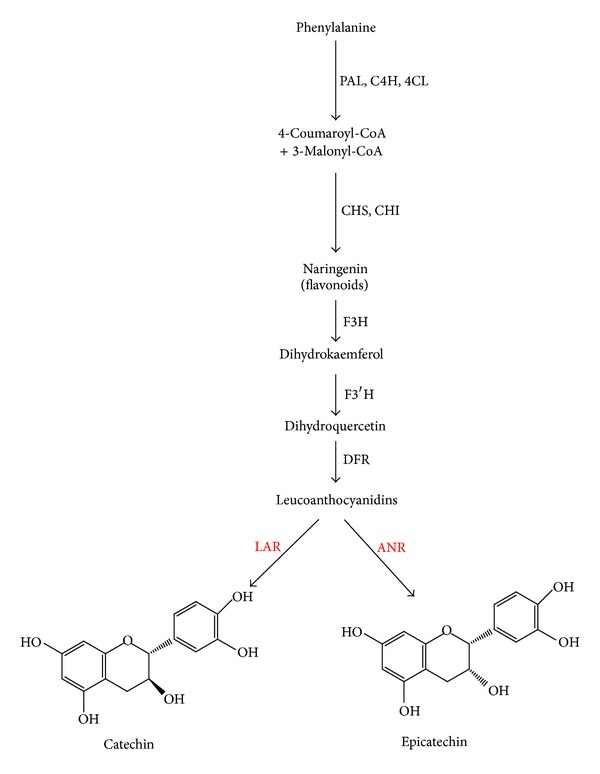
Flavonoid biosynthesis pathway in *F. tataricum*. PAL, phenylalanine ammonia lyase; C4H, cinnamate 4-hydroxylase; 4CL, 4-coumaroyl CoA ligase; CHS, chalcone synthase; CHI, chalcone isomerase; F3H, flavones 3-hydroxylase; F3′H, flavonoid 3′-hydroxylase; DFR, dihydroflavonol-4 reductase; ANR, anthocyanidin reductase; LAR, leucoanthocyanidin reductase. The red color genes were isolated from *F. tataricum* in this study.

Proanthocyanidins (PAs; also known as condensed tannins) are phenolic oligomers or polymers that result from the polymerization of flavan-3-ol units; they are synthesized from the first metabolites via the shikimate and flavonoid pathway [1, 2]. PAs provide multiple health benefits to humans, such as antioxidant, anticancer, and anticardiovascular effects [3–5]. PAs are one of the final products of the flavonoid pathway and contribute to the quality of many important plant products, such as wine, tea, and cocoa [6]. Winkel-Shirley [7] reported that PAs are derived from the pathway leading to anthocyanins, a class of flavonoids well understood at both the biochemical and molecular genetic levels. Leucoanthocyanidin reductase (LAR) and anthocyanidin reductase (ANR) are both key enzymes of the branch pathway of PAs biosynthesis (Figure 1). ANR is one of two enzymes of the flavonoid biosynthesis pathway that produces flavan-3-ol (epicatechin) monomers, producing epicatechin from anthocyanidin [8]. ANR was initially found to be encoded by the BANYULS (BAN) gene from *Arabidopsis thaliana *and* Medicago truncatula* [9]. ANR enzymatic function has been identified in various plants, such as grape (*Vitis vinifera*), soybean (*Glycine max*), tea (*Camellia sinensis*), and legume (*Medicago truncatula*) [10–13]. Bogs et al. [10] reported that LAR catalyzes the conversion of leucocyanidin to catechin, clearly establishing its role in PA biosynthesis. The functionality of LAR has been reported in several plants and its activity is correlated with PA accumulation [11, 13, 14].

Buckwheat (*Fagopyrum esculentum *and* Fagopyrum tataricum*), a dicotyledonous crop of the Polygonaceae family, has received attention as health food. In particular, tartary buckwheat has also been found to have several beneficial pharmacological and biological effects, such as anticancer, antidiabetic, and antioxidant activities [15–17]. In addition, tartary buckwheat is a richer source of rutin, a flavonol glycoside that prevents ultraviolet light-induced DNA damage and disease, than common buckwheat [16, 17]. Recent research on buckwheat has focused on functional food material, particularly with respect to seed sprouts in Korea and Japan. Tartary buckwheat sprouts are an excellent dietary source of phenolic compounds [17]. The sprouts of the *F. tataricum *“Hokkai T10” (T10) cultivar, derived from “Hokkai T8” (T8) by chemical treatment (ethyl methane sulfonate), have a higher anthocyanin content than common buckwheat [18]. The duration and amount of light exposure that was used for sprouting strongly affect the nutritional quality of tartary buckwheat sprouts [19].

When common buckwheat sprouts (*F. esculentum*) were grown under light or dark condition, flavonoid content was increased significantly [20]. In present study, we cloned ANR and LAR genes involved in PAs biosynthesis and examined the expression of these two genes in different organs under different growth conditions in two tartary buckwheat cultivars, Hokkai T8 and T10. In addition, we analyzed content of catechin and epicatechin in different organs and sprouts.

## 2. Materials and Methods

### 2.1. Plant Materials and Growth Conditions

Two tartary buckwheat cultivars, T8 and T10, were bred by the Hokkaido Agricultural Research Center (Hokkaido, Japan). The seeds were surface-sterilized with 70% ethanol for 1 min and 4% (v/v) bleach solution for 20 min and then rinsed several times in sterile water. Sterilized seeds were germinated on 1/2 MS medium in a growth chamber under light condition (16 h light/8 h darkness) and dark condition (24 h darkness) at 25°C, 60% humidity, and 440 *μ*moles/m^2^/s light intensity. For biological replicates, we used 3 plastic boxes for each single treatment placing 30 seeds per box. Sprouts including roots were harvested at 0, 3, 6, 9, and 12 days after sowing DAS. The seeds of both T8 and T10 were sown on 10 May, 2012, and then transferred into pots filled with the perlite-mixed soil. Tartary buckwheat plants were grown in the greenhouse (25°C and 50% humidity) at Chungnam National University (Daejeon, Korea). Different plant organs (seed stages 1, 2, and 3, flowers, stems, leaves, and roots) were collected after 6 weeks. The seed stages were distinguished as described previously [21]. All samples were frozen in liquid nitrogen upon collection and stored at −80°C until use.

### 2.2. Isolation of Genes Encoding ANR and LAR from *F. tataricum*

The full-length cDNA sequence of putative ANR was obtained from next generation sequencing platforms (NGS) (Roche/454 GS_FLX+ and Illumina/Solexa HiSeq2000) (unpublished data) of *F. tataricum* of *SolGent* company (Daejeon, Korea). LAR partial sequences were obtained from NGS data and a full-length cDNA was obtained using rapid amplification of cDNA ends (RACE) PCR. Sequence similarities were calculated with the Basic Local Alignment Search Tool (BLAST) (http://blast.ncbi.nlm.nih.gov/). The sequence data were used to design new primer pairs for RACE PCR, qRT-PCR, and ORF PCR, and the primes were mentioned in Supplementary Table  S1 (see Supplementary Material available online at http://dx.doi.org/10.1155/2014/726567).

### 2.3. Total RNA Extraction and cDNA Synthesis

Total RNA was isolated from two tartary buckwheat sprouts and different organs. The total RNA of sprouts was isolated using the RNeasy Plant Mini Kit (Qiagen, Valencia, CA), whereas that from different organs was extracted by a modified CTAB (cetyltrimethylammonium bromide) method because of complex polysaccharides [22]. The RNA pellet was washed with 70% EtOH and dissolved in DEPC water. RNA quantity and quality were determined by a NanoVue Plus Spectrophotometer (GE Health Care Life Sciences, USA) and assessed by running 1 *μ*g of total RNA on 1.2% formaldehyde RNA agarose gel, respectively. Subsequently, 1 *μ*g of total RNA was reverse-transcribed using the ReverTra Ace-*α*-(Toyobo, Osaka, Japan) Kit and oligo (dT)_20_ primer according to the manufacturer's protocol. The synthesized cDNA was used as the template for qRT-PCR and RACE PCR.

### 2.4. Quantitative Real-Time RT-PCR Analysis

For qRT-PCR, the TM calculator program (http://bioinfo.ut.ee/primer3-0.4.0/) was used to compute the PCR annealing temperatures. qRT-PCR assay was carried out in a total volume of 20 *μ*L, containing 10 *μ*L of 2 X SYBR Green Real-time PCR master mixes (Toyobo, Osaka, Japan), 0.5 *μ*M (each) of specific primers, and 5 *μ*L of cDNA that was diluted 20-fold. The amplification program consisted of one cycle of 95°C for 3 min, followed by 40 cycles of 95°C for 15 s, 72°C for 20 s, and annealing temperature 55°C for 30 s. The reaction was performed in triplicate on a CFX96 Real-Time PCR System (Bio-Rad; Hercules, CA, USA). The histone H3 gene (GenBank number HM628903) was used as a reference gene [19–21].

### 2.5. Bioinformatic Analysis of FtANR and FtLAR

Alignment of the deduced amino acid sequences of FtANR and FtLAR was carried out using the Biological Sequence Alignment Editor (BioEdit) software. The phylogenetic relationships of FtANR and FtLAR were analyzed using ClustalX and MEGA version 4.0. In the bootstrap, the multiple alignment was resampled 100 times. Theoretical molecular weights and pI values were calculated by the Compute pI/Mw tool (http://ca.expasy.org/tools/pi_tool.html). The secondary structure was predicted using SOPMA (http://npsa-pbil.ibcp.fr/cgi-bin/npsa_automat.pl?page=/NPSA/npsa_sopma.html). The putative target location of the plant was predicted online through PSORT (http://wolfpsort.org/).

### 2.6. Estimation of Catechin and Epicatechin

Chemical analysis of catechin and epicatechin was carried out by HPLC analysis and we used a minor modification of a previously published method [23]. For this, freeze-dried samples of buckwheat sprouts and different organs were ground into a fine powder using a mortar and pestle. Powdered samples (~100 mg) were extracted with 80% (v/v) methanol at room temperature for 60 min. Subsequently, the extracts were centrifuged, and the supernatant was filtered with a 0.45 *μ*m Acrodisc syringe filter (Pall Corp.; Port Washington, NY) for HPLC. HPLC analysis was performed with a C18 column (*μ*Bondapak C18 10 *μ*m, 125 Å, 3.9 × 300 mm). The mobile phase was a gradient pattern prepared from mixtures of methanol and 0.5% acetic acid. The flow rate was maintained at 0.8 mL/min. An injection volume of 20 *μ*L and wavelength of 280 nm were used for detection. The compounds in the sample were determined using a standard curve. All samples were analyzed in triplicate.

## 3. Results and Discussion

### 3.1. Isolation and Sequence Analysis of FtANR and FtLAR from Tartary Buckwheat

Open reading frame (ORF) of FtANR (Genbank Accession number KC404848) obtained from NGS data for tartary buckwheat was 1011 bp long, encoding a protein of 336 amino acids with a theoretical molecular weight of 36.8 kDa and a pI value of 5.42. *Medicago truncatula* (MtANR) and *Arabidopsis thaliana *(AtANR) consisted of 338 and 340 amino acids with molecular weights of 36.9 and 37.9 kDa, respectively [24]. From BLAST analysis of the ANR-deduced amino acid sequences, FtANR was found to share 73%, 77%, 82%, 78%, and 80% identities with *M. truncatula *(AAN77735)*, Malus *× *domestica *(JN035299)*, Gossypium hirsutum *(EF187443),* Camellia sinensis *(AAT68773), and* V. vinifera* (BAD89742), respectively. FtANR was found to have a conserved motif GXGXXA similar to that found in monodehydroascorbate reductase (NADH) (Supplementary Figure S1) [25].

Using RACE technology with partial sequences obtained from NGS data, we isolated 1581-bp-long FtLAR cDNA (Genbank accession number KC404849) from flowers of *F. tataricum* T10, which contained a 1176-bp ORF; this gene encoded a protein of 391 amino acids with a theoretical molecular mass of 43.3 kDa and a pI value of 5.27. Recently, Ma et al. [26] cloned and characterized the LAR gene from *Fagopyrum dibotrys*. The amino acid sequences of FtLAR and FdLAR were found to differ by only 12 amino acids. The FdLAR target fusion peptide had a molecular weight of 66 kDa [26], which did not correspond to the theoretical molecular weight of FtLAR. The amino acid sequence of FtLAR showed 68%, 70%, 69%, and 66% identity with *V. vinifera* (AAZ82410), *Prunus avium* (ADY15310), *Diospyros kaki* (BAH89267), and *Malus *×* domestica* (AAX12186), respectively. In particular, FtLAR showed 97% homology with *F. dibotrys* (JN793953). As reported previously [10, 27], FtLAR had specific amino acid motifs of ICCN and THD (Supplementary Figure S2). The subcellular targeting of FtANR was predicted to be in the cytosol and the chloroplast, whereas FtLAR was predicted to be localized to the cytosol. This result was in accordance with that of a previous study [11]. Winkel [28] suggested that the PA pathway exists as a metabolic channel associated with cellular membranes.

**Figure 2:**
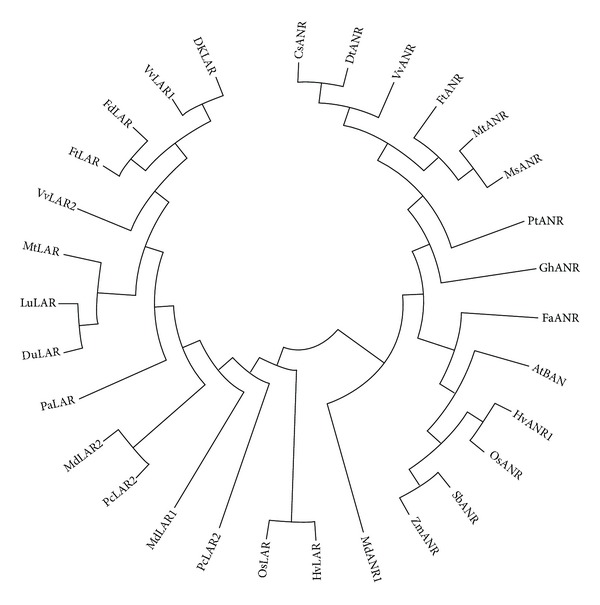
Phylogenetic relationships of ANR and LAR proteins from *F. tataricum* and select species. GenBank accession numbers are AtBAN (*Arabidopsis thaliana*, NP_176365), CsANR (*Camellia sinensis*, AAT68773), MtANR (Medicago truncatula, AAN77735), VvANR (*Vitis vinifera,* BAD89742), FaANR (*Fragaria *×* ananassa*, DQ664192), PtANR (*Populus trichocarpa*, XM_002317234), GhANR (*Gossypium hirsutum*, EF187443), DkANR (*Diospyros kaki*, AB195284), MsANR (*Medicago sativa*, HM754630), HvANR1 (*Hordeum vulgare*, AK373696), OsANR (*Oryza sativa*, NM_001060512), SbANR (*Sorghum bicolor*, XM_002447113), ZmANR (*Zea mays*, BT064433), MdANR1 (*Malus *×* domestica*, JN035299), FtANR (*Fagopyrum tataricum*, KC404848). DuLAR (*Desmodium uncinatum*, CAD79341), LuLAR (*Lotus uliginosus*, AAU45392), VvLAR1 (*Vitis vinifera*, AAZ82410), VvLAR2 (*Vitis vinifera*, AAZ82411), HvLAR (*Hordeum vulgare*, CAI56320), OsLAR (*Oryza sativa*, CAI56328), MdLAR1 (*Malus *×* domestica*, AAX12185), MdLAR2 (*Malus *×* domestica*, AAX12186), MtLAR (*Medicago truncatula*, CAI56327), PcLAR1 (*Phaseolus coccineus*, CAI56322), PcLAR2 (*Phaseolus coccineus*, CAD91909), DkLAR (*Diospyros kaki*, BAH89267), PaLAR (*Prunus avium*, ADY15310), FdLAR (*Fagopyrum dibotrys*, AEY62396), and FtLAR (*Fagopyrum tataricum*, KC404849).

To determine the relationship of putative FtANR and FtLAR proteins with other plant ANRs and LARs, we performed phylogenetic analysis (Figure 2). As described previously [29], a phylogenetic tree of FtANR was clustered into 2 distinct groups, monocot and dicot species. FtANR is most closely related to *V. vinifera*, while FtLAR is most closely related to *F. dibotrys.* Like the ANR protein, LAR proteins were separated into monocot (*Hordeum vulgare *and* Oryza sativa*) and dicot species.

### 3.2. Gene Expression of *FtANR* and *FtLAR* during Sprout Development under Dark and Light Conditions in T8 and T10

**Figure 3:**
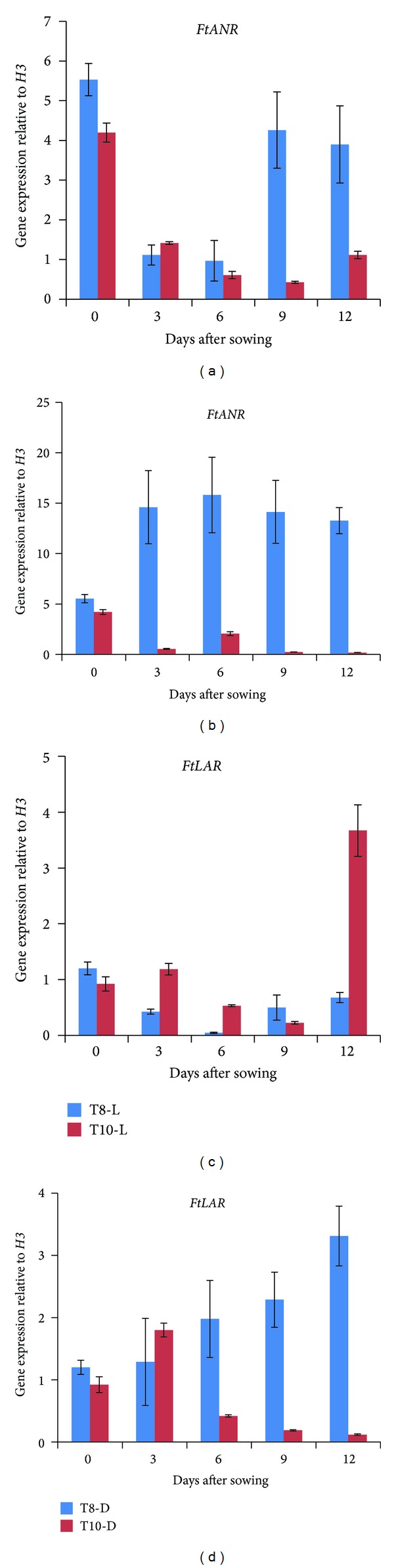
Relative expression levels of *FtANR *and* FtLAR* in seedling of *F. tataricum* T8 and T10 cultivars. L-light condition, D-dark condition. The height of each bar and the error bars show the mean and standard error, respectively, from 3 independent measurements.

Transcript levels of ANR and LAR in seedlings of T8 and T10 were traced at 3 d intervals from 0 to 12 d after sowing (DAS) under dark and light conditions by real-time RT-PCR analysis (Figure 3). Seeds were considered as 0 DAS in this study. Generally, *FtANR* expression levels were higher in T8 than T10 under both conditions. Specifically, transcript level in T8 under dark condition was higher than that under light condition, although fluctuation pattern was observed in light condition. In particular, *FtANR* transcript levels were the highest at 0 day and sharply declined in 3 and 6 DAS for both T8 and T10 cultivars under light condition. However, *FtANR *expression was increased from 0 to 3 DAS for T8 cultivar under dark condition and remained relatively constant on 6, 9, and 12 DAS. Unlike *FtANR*, different gene expression pattern was observed in *FtLAR.* The highest gene expression level was observed at 12 DAS in T10 which was nearly 5 times higher than that in T8 under light condition. In addition, a gradual increase in transcript levels was observed in T8 under dark condition which is similar as *FtANR* under dark condition. Therefore, in this study, the transcription of *FtANR *and* FtLAR* in T8 and T10 sprouts was unaffected by light conditions.

### 3.3. Gene Expression of *FtANR* and *FtLAR* among Different Organs of T8 and T10

**Figure 4:**
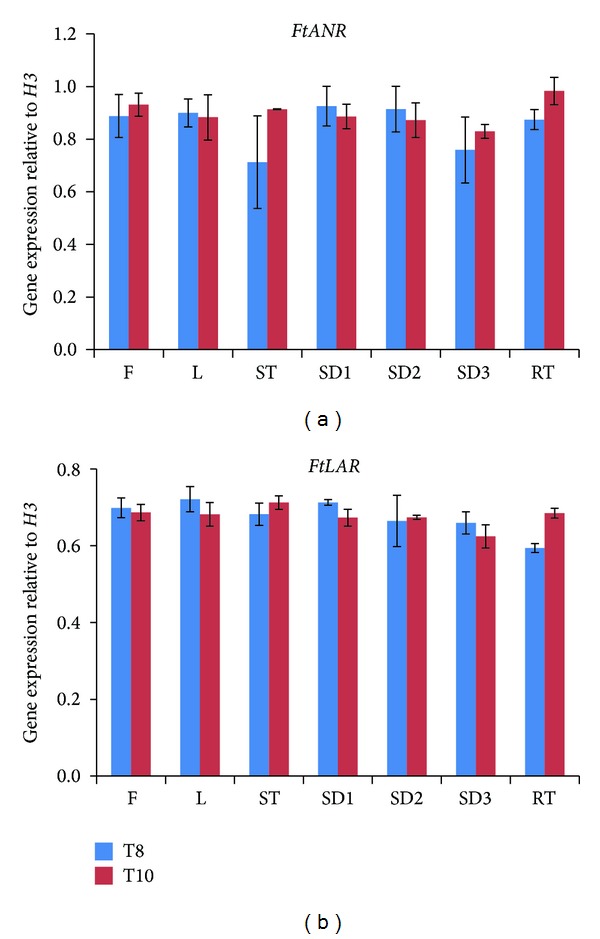
Relative expression levels of *FtANR *and* FtLAR* in different organs of *F. tataricum* T8 and T10 cultivars. F, flowers; ST, stems; L, leaves; SD 1, 2, 3, seed stage 1, 2, 3; RT, roots. The height of each bar and the error bars show the mean and standard error, respectively, from 3 independent measurements.

The mRNA levels of *FtANR* and *FtLAR *among different organs (flower, leaf, stem, root, and seeds in stages 1, 2, and 3) were shown in Figure 4. Unlike transcript levels during sprout development, *FtANR* and *FtLAR *did not show large variations in their expression patterns among all the organs in T8 and T10. In some apple species, *LAR1* expression was similar in the cortex and skin, while ANR expression levels were similar in apple skin flesh [30]. The relative levels of *P. trichocarpa LAR3* transcripts in the roots were 2- and 3-fold higher than those in the stems and petioles, respectively [31]. Bogs et al. [10] reported that the two LAR genes involved in PA biosynthesis had different patterns of expression in grape seeds and skins, which affect the concentration and composition of PAs. They suggested that *V. vinifera* ANR may have a more significant role in PAs synthesis in grapevine leaves than LAR. In addition, *Glycine max ANR1* is predominantly expressed in the seed coat, whereas *GmANR2* is expressed at low levels in all organs [29]. Devic et al. [32] reported that the expression of the BAN gene is limited to the endothelium of immature seeds in *Arabidopsis*, whereas in *Medicago*, its expression is seen in young seeds, flowers, and leaves [9]. Recently, it was reported that overexpression of LAR3 in Chinese white poplar (*Populus tomentosa*) leads to plantwide increase in PA levels that is the highest in the roots [31]. In our study, we found that the transcript levels of *FtANR* and *FtLAR *were similar among different organs. According to the transcript levels observed in other plants, it appears that FtANR and FtLAR are regulated differently compared to other plant species. It is also possible that FtANR and FtLAR isoforms may exist, and these isoforms may contribute to catechin and epicatechin biosynthesis observed in this study in T8 and T10 tartary buckwheat.

### 3.4. Analysis of Catechin and Epicatechin during Seedling Development of Tartary Buckwheat

**Figure 5:**
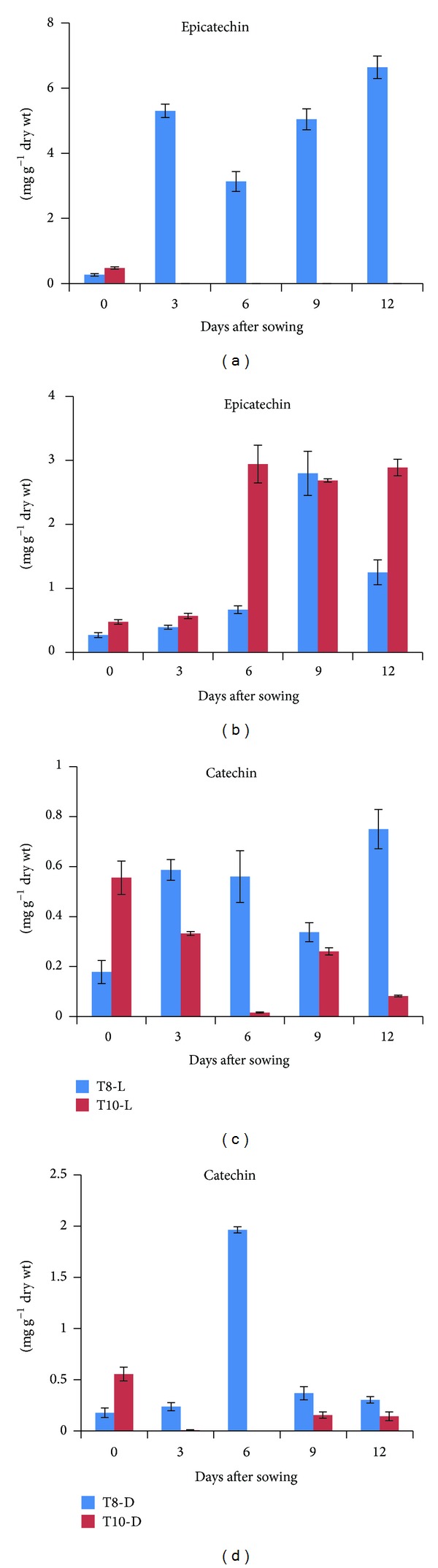
Epicatechin and catechin content in seedling of *F. tataricum* T8 and T10 cultivars. (a), (c) light condition; (b), (d) dark condition. The height of each bar and the error bars show the mean and standard error, respectively, from 3 independent measurements.

The composition of catechin and epicatechin during seedling development from T8 and T10 was determined by HPLC (Figure 5). Epicatechin was not detected in T10 sprouts under light conditions, whereas T8 sprouts had a high level of epicatechin (Figure 5(a)). The epicatechin content of T8 was the highest (6.6 mg g^−1^ dry weight [DW]) at 12 DAS under light conditions. The opposite pattern was observed under dark conditions, where the epicatechin content of T10 was higher than that of T8. Under dark conditions, the epicatechin content of T10 was low in seeds and at 3 DAS but reached higher levels on 6, 9, and 12 DAS (e.g. 0.47 mg g^−1^ DW Day 0 and 2.9 mg g^−1^ DW on 6 DAS), while that of T8 increased from 0 (0.27 mg g^−1^ DW) to 9 DAS (2.79 mg g^−1^ DW) (Figure 5(b)). Under light conditions, the epicatechin content of T8 increased gradually from 6 to 12 DAS. The epicatechin content of T8 was the highest (6.6 mg g^−1^ DW) at 12 DAS under light conditions, while T10 exhibited the highest content (3.1 mg g^−1^ DW) at 6 DAS under dark conditions.

The catechin content of T8 under light and dark conditions was higher than that of T10 (Figures 5(c) and 5(d)). The catechin content of T8 was the highest at 6 DAS (1.96 mg g^−1^ DW) and 12 DAS (0.75 mg g^−1^ DW) under dark and light conditions. The catechin content of T10 was the highest at 0 DAS (0.55 mg g^−1^ DW). Under dark conditions, the catechin content of T8 increased from 0 (0.17 mg g^−1^ DW) to 6 DAS (1.96 mg g^−1^ DW) and then decreased to the levels similar to that of Day 0. On the other hand, under light conditions, the catechin content of T10 decreased gradually from 0 (0.55 mg g^−1^ DW) to 6 DAS (0.015 mg g^−1^ DW). Under light conditions, the catechin content of T8 was 37- and 9-fold higher at 6 and 12 DAS, respectively, than in T10.

Unlike catechin analysis, epicatechin content was affected by light in T8 only, whereas T10 remained unaffected by light. In general, light acts as an essential stimulus and also modulates the intensity of the pigment by affecting the regulatory and structural genes involved in anthocyanin biosynthesis [33]. However, Bakhshi and Arakawa [34] described that the content of phenolic acids, anthocyanin, and flavonols increased rapidly by irradiation, whereas flavanols, procyanidins, and dihydrochalcones did not change in either mature or in ripe apple fruits. Our results suggest that a response of catechin and epicatechin content to light might depend on the tartary buckwheat cultivar.

### 3.5. Analysis of Catechin and Epicatechin in Different Organs

**Figure 6:**
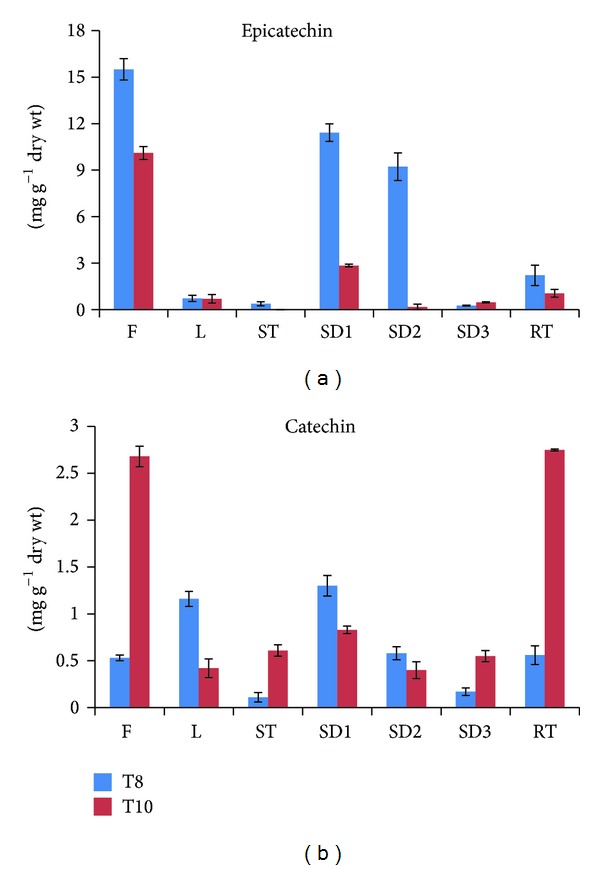
Epicatechin and catechin content in different organs of *F. tataricum* T8 and T10 cultivars. The height of each bar and the error bars show the mean and standard error, respectively, from 3 independent measurements.

Catechin and epicatechin contents were analyzed from different organs from T8 and T10 (i.e., flowers, stems, leave, roots, and seeds at stages 1, 2, and 3) by HPLC (Figure 6). Similar to the results seen in seedling development, epicatechin content was higher than the catechin content in both T8 and T10. In T10, catechin was found mostly in the flowers (2.68 mg g^−1^ DW) and roots (2.75 mg g^−1^ DW), whereas in T8, higher amounts were found in leaves (1.16 mg g^−1^ DW) and seeds at stage 1 (1.3 mg g^−1^ DW). It is interesting that catechin content in T10 was the highest in the roots. T10 wild roots in the soil and hairy roots are red in color. These results indicate that catechin content is correlated with color pigment. The lowest catechin content was found in the stems (0.11 mg g^−1^ DW) of T8 and in seeds at stage 2 (0.4 mg g^−1^ DW) of T10. The catechin content of seeds decreased gradually from stage 1 (1.3 mg g^−1^ DW) to stage 3 (0.17 mg g^−1^ DW).

The flowers of T8 and T10 contained the highest levels of epicatechin (15.5 and 10.1 mg g^−1^ DW, resp.), whereas the lowest amount of epicatechin was in the seeds at stage 3 in T8 (0.3 mg g^−1^ DW). However, in all cases, T8 exhibited higher amounts of epicatechin content than T10, and in fact, epicatechin was not detected in the stems of T10. The epicatechin content in T8 flowers was 22-fold higher than that in their leaves. Similarly, in T10 flowers, the level of epicatechin was 14-fold higher than in leaves of the same cultivar. Li et al. [20] reported that in common buckwheat, the catechin hydrate and epicatechin content in flowers is higher than in other organs. The results from the present study indicate that catechin and epicatechin accumulated abundantly in the organs of both cultivars and were found more abundantly in the flowers, thus supporting a previous report by Uddin et al. [35] wherein the flower of the common buckwheat was found to contain many more phenolic compounds than other plant parts. Watanabe [23] reported that antioxidant activity of catechins isolated from common buckwheat groats was superior to that of rutin, which is known as an antioxidant in buckwheat, at the same concentration. Further study is required for antioxidant activity of catechins and epicatechin isolated from tartary buckwheat cultivars, T8 and T10.

## 4. Conclusion

Our study shows that epicatechin content is affected by light in both T8 and T10. We observed that catechin and epicatechin content varies in cultivars. The highest amount of epicatechin was observed in the flowers of both tartary buckwheat cultivars. The highest amount of epicatechin was observed in the flowers of both tartary buckwheat cultivars we studied. These data suggest that the two types of tartary buckwheat sprouts may have different mechanisms for catechin and epicatechin biosynthesis, which vary under light and dark conditions. The data obtained in our study provides evidence that cultivar differences as well as differences in environmental conditions can differentially regulate flavonoid biosynthesis.

## Supplementary Material

Table S1: Primers used in this study.
Figure S1: Sequence alignment of the deduced FtANR sequence with other plant ANR sequences. Sequences are from AtBAN (*Arabidopsis thaliana*, NP_176365), CsANR (*Camellia sinensis*, AAT68773), MtANR (*Medicago truncatula*, AAN77735), VvANR (*Vitis vinifera*, BAD89742), ZmANR (*Zea mays*, BT064433), and FtANR (*Fagopyrum tataricum*, KC404848). Identical amino acids are indicated by white letters on a black background. A thick red box and asterisks above the sequence indicate NADPH binding site and the catalystic triad, respectively. Peptide sequences were aligned with the BioEdit program.
Figure S2: Sequence alignment of the deduced FtLAR sequence with other plant LAR sequences. Sequences are from VvLAR1 (*Vitis vinifera*, AAZ82410), MtLAR (*Medicago truncatula*, CAI56327), FdLAR (*Fagopyrum dibotrys*, AEY62396), MdLAR1 (*Malus* x *domestica*, AAX12185), and FtLAR (*Fagopyrum tataricum*, KC404849). Identical amino acids are indicated by white letters on a black background. Asterisks show the RFLP, ICCN, and THD motifs. Peptide sequences were aligned with the BioEdit program.

## Abbreviations

ANR: Anthocyanidin reductase
LAR: Leucoanthocyanidin reductase
HPLC: High-performance liquid chromatography
qRT-PCR: Quantitative real-time RT-PCR.
