# Supplementary material for: Transcripts of Anthocyanidin Reductase and Leucoanthocyanidin Reductase and Measurement of Catechin and Epicatechin in Tartary Buckwheat
Source: ScientificWorldJournal. 2014 Jan 27;2014:726567. doi: 10.1155/2014/726567 (PMC3926278; doi:10.1155/2014/726567)
Supplement: Supplementary file 1 — Table S1: Primers used in this study. Figure S1: Sequence alignment of the deduced FtANR sequence with other plant ANR sequences. Sequences are from AtBAN (Arabidopsis thaliana, NP_176365), CsANR (Camellia sinensis, AAT68773), MtANR (Medicago truncatula, AAN77735), VvANR (Vitis vinifera, BAD89742), ZmANR (Zea mays, BT064433), and FtANR (Fagopyrum tataricum, KC404848). Identical amino acids are indicated by white letters on a black background. A thick red box and asterisks above the sequence indicate NADPH binding site and the catalystic triad, respectively. Peptide sequences were aligned with the BioEdit program. Figure S2: Sequence alignment of the deduced FtLAR sequence with other plant LAR sequences. Sequences are from VvLAR1 (Vitis vinifera, AAZ82410), MtLAR (Medicago truncatula, CAI56327), FdLAR (Fagopyrum dibotrys, AEY62396), MdLAR1 (Malus x domestica, AAX12185), and FtLAR (Fagopyrum tataricum, KC404849). Identical amino acids are indicated by white letters on a black background. Asterisks show the RFLP, ICCN, and THD motifs. Peptide sequences were aligned with the BioEdit program. [file 726567.f1.docx]

**Supplementary Data**

TABLE S1: Primers used in this study.

| Use | Primer name | Sequence (5' → 3') |
| --- | --- | --- |
| RACE PCR | FtLAR-3’RACE (F) | CGGTGCGACGGGCGATAGAGGA |
|  | FtLAR-5’RACE (R) | ATCGTAGTACGGCCAAGATGCTATGGAGTTGCAG |
| ORF | FtANR-ORF (F) | ATGGCTGCCCAAGTTGGTG |
|  | FtANR-ORF (R) | CTAGTTCTTTAGCATACCAACTTCCTTCA |
|  | FtLAR-ORF1 (F1) | ATGACTGTTG CCGTGACCGC CATCC |
|  | FtLAR-ORF1 (R1) | TCAACACACTGCAGGGACTGGTAAGGCC |
| Real-time PCR | FtANR-RT (F1) | ATGACCGGCCGTTCTATTAC |
|  | FtANR-RT (R1) | CCAGACAGCATTTGCATACC |
|  | FtLAR-RT(F) | CGATGTACTTGGAGAAGCGA |
|  | FtLAR-RT(R) | GGTCCATAGGAGGTGTGACC |
|  | FeH3-RT(F) | GAAATTCGCAAGTACCAGAAGAG |
|  | FeH3-RT(R) | CCAACAAGGTATGCCTCAGC |

**
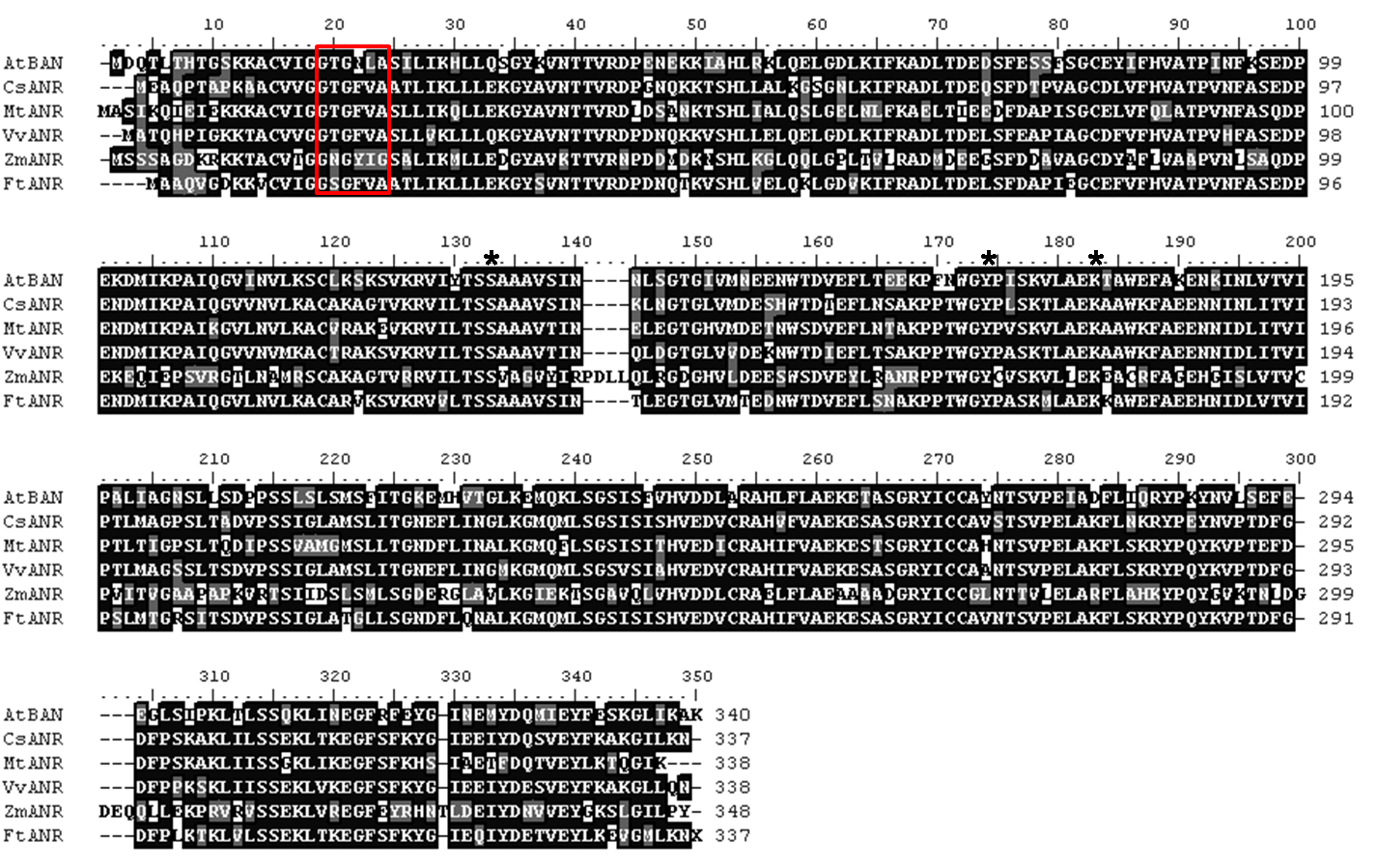
**

FIGURE S1: Sequence alignment of the deduced FtANR sequence with other plant ANR sequences. Sequences are from AtBAN (*Arabidopsis thaliana*, NP_176365), CsANR (*Camellia sinensis*, AAT68773), MtANR (*Medicago truncatula*, AAN77735), VvANR (*Vitis vinifera*, BAD89742), ZmANR (*Zea mays*, BT064433), and FtANR (*Fagopyrum tataricum*, KC404848). Identical amino acids are indicated by white letters on a black background. A thick red box and asterisks above the sequence indicate NADPH binding site and the catalystic triad, respectively. Peptide sequences were aligned with the BioEdit program.


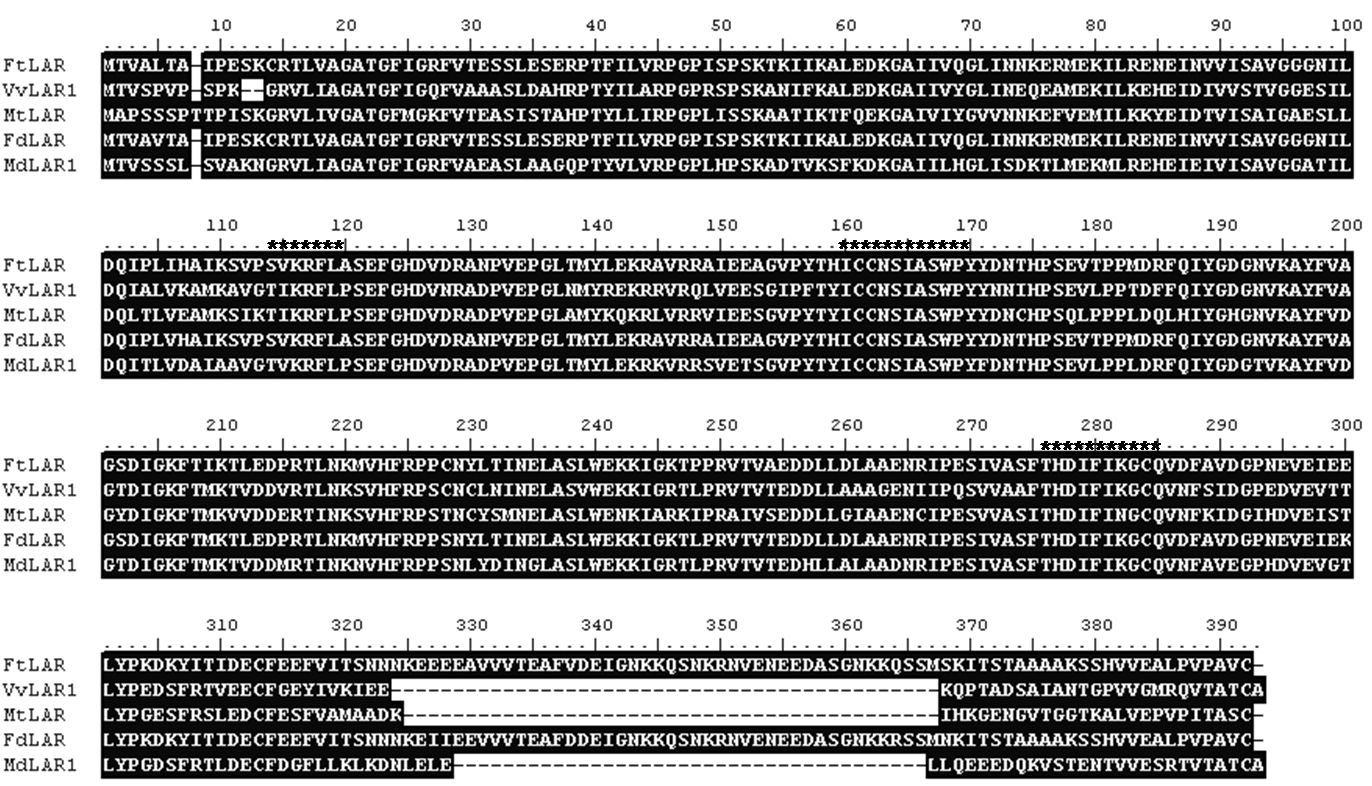


FIGURE S2: Sequence alignment of the deduced FtLAR sequence with other plant LAR sequences. Sequences are from VvLAR1 (*Vitis vinifera*, AAZ82410), MtLAR (*Medicago truncatula*, CAI56327), FdLAR (*Fagopyrum dibotrys*, AEY62396), MdLAR1 (*Malus* x *domestica*, AAX12185), and FtLAR (*Fagopyrum tataricum*, KC404849). Identical amino acids are indicated by white letters on a black background. Asterisks show the RFLP, ICCN, and THD motifs. Peptide sequences were aligned with the BioEdit program.
